# P-848. Antibiotic Prescribing in VA Healthcare System Emergency Departments and Urgent Care Centers – a Medication Use Evaluation

**DOI:** 10.1093/ofid/ofaf695.1056

**Published:** 2026-01-11

**Authors:** Kristina L Bajema, Judith Strymish, Makoto M Jones, Muriel Burk, Madeline McCarren, Fran Cunningham, Michael Lorenz, Samaneh Ghassemi, Anna Chen, Yu Wang, Kelly Echeverria, Karl Madaras-Kelly, Matthew B Goetz

**Affiliations:** Veterans Affairs Portland Health Care System, Oregon Health and Science University, Portland, Oregon; VA Boston Healthcare System, West Roxbury, MA; Veterans Affairs, Salt Lake City, Utah; VA PBM Center for Medication Safety, Hines, Illinois; Dept of Veterans Affairs, Hines, Illinois; US Dept of Veterans Affairs, Hines, Illinois; VA St. Louis HCS, St. Louis, Missouri; Edward Hines Jr. VA, Hines, Illinois; VA Boston Healthcare System, West Roxbury, MA; VA PBM, Chicago, Illinois; Retired, San Antonio, Texas; Idaho State University, Boise, Idaho; VA Greater Los Angeles Healthcare System, Los Angeles, California

## Abstract

**Background:**

Inappropriate antibiotic selection &/or duration has been reported in 40–65% of patients treated in the Emergency Department (ED). Antimicrobial stewardship program (ASP) interventions in the ED are challenged by time constraints and diagnostic uncertainty. Most studies of ED antibiotic use rely on ICD-10 diagnostic codes, not clinical chart review.

**Figure 1.**
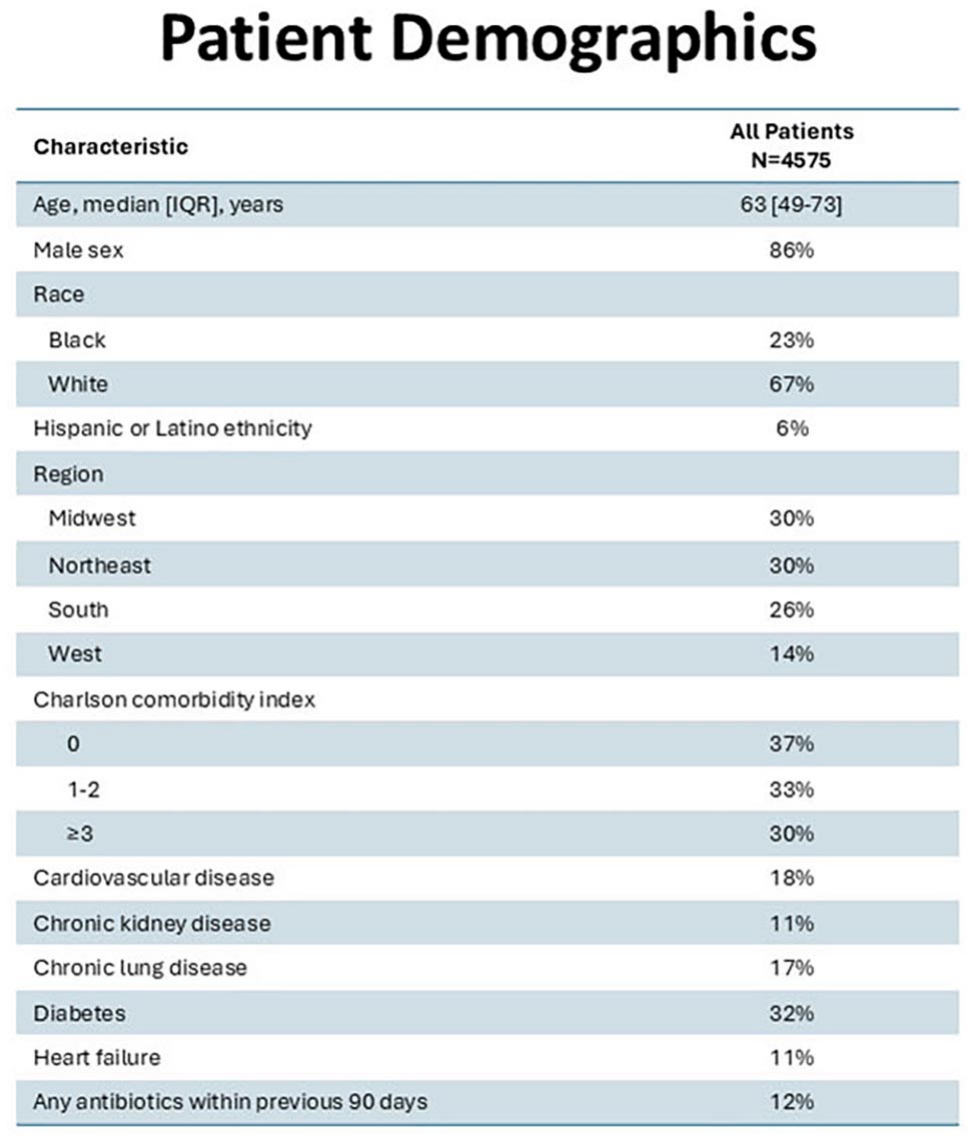
Patient Demographics

**Figure 2.**
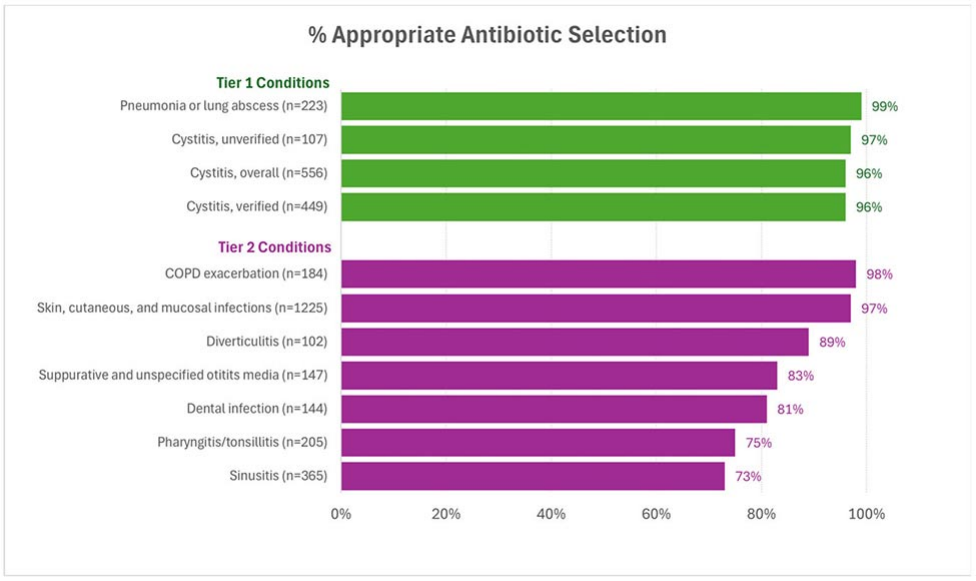
Appropriateness of Antibiotic Selection Prevalence of Inappropriate Antibiotic Prescriptions Among US Ambulatory Care Visits, 2010-2011 | Infectious Diseases | JAMA | JAMA Network, Measurement and Evaluation Approaches to Improve Outpatient Antibiotic Prescribing in Health Systems

**Methods:**

We conducted a Medication Use Evaluation (MUE) at 40 Veterans Affairs facilities to assess oral antibiotic prescribing in ED & urgent settings. Objectives were to: 1) assess antibiotic prescribing using CDC diagnostic tiers—Tier 1 (almost always indicated), Tier 2 (sometimes indicated), & Tier 3 (rarely indicated); 2) determine the appropriateness of antibiotic selection and duration based on national guidelines; & 3) compare chart documentation with ICD-10 codes.

Each site reviewed ∼100 eligible records using a standardized MUE protocol. Patients were included if they received an oral antibiotic prescription within 24 hours of an ED or urgent care visit from June 1, 2022 to May 31, 2023. Exclusions included receipt of antibiotics or hospitalization within 7 days prior to the encounter. MUE was determined as non-research by Hines IRB.

**Figure 3.**
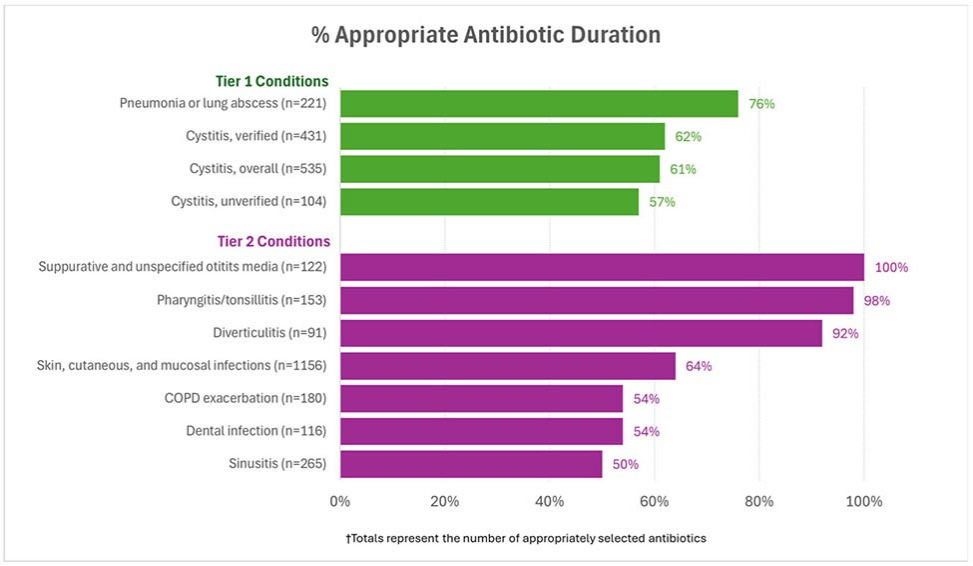
Duration of Antibiotic Prescribing among Persons with Appropriate Antibiotic Selection

**Figure:**
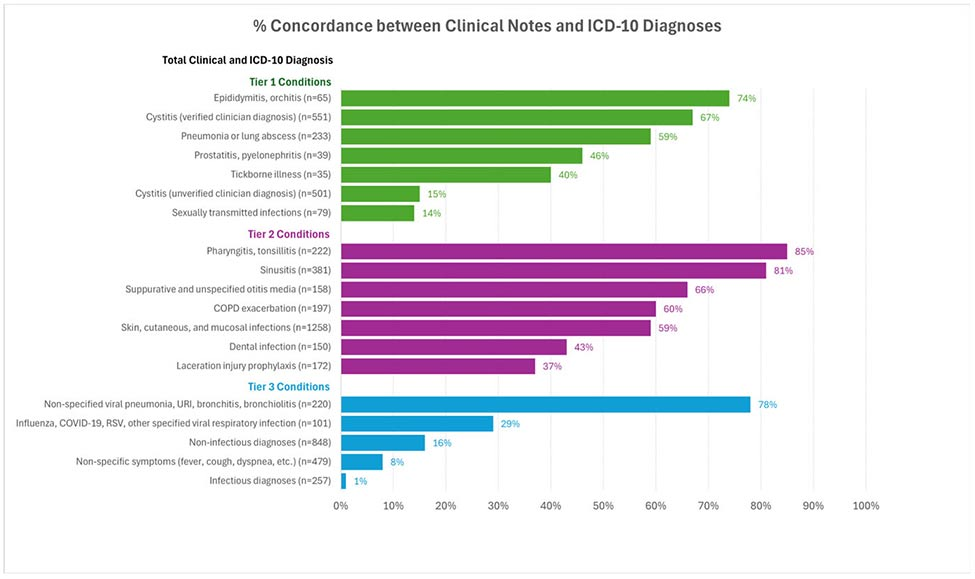
Concordance of Clinical Notes and ICD-10 Diagnoses (%)

**Results:**

Among 4575 Veterans included in the evaluation (Figure 1), clinician documentation indicated that 22%, 59% & 19% had Tier 1, 2 & 3 diagnoses, respectively. Appropriateness of antibiotic selection (Figure 2) was >80% except for sinusitis (73%) & pharyngitis (75%). Appropriateness of antibiotic duration (Figure 3) was 76% for pneumonia & < 65% for sinusitis, SSTIs, dental infections, and cystitis. Tier 3 durations often exceeded 5 days (Figure 2). Concordance between clinical notes and ICD-10 codes was poor (46%) across all tiers (Figure 4).

**Conclusion:**

Improving stewardship in EDs will benefit from multidisciplinary effort with engagement of ED practitioners, pharmacists & ASPs. This MUE highlights opportunities to reduce Tier 3 prescribing, develop standardized infection treatment guidelines, & improve documentation & coding accuracy. The high level of discordance between chart review & ICD-10 coded diagnoses underscores the challenges in using administrative data to assess antibiotic prescribing appropriateness.

**Disclosures:**

All Authors: No reported disclosures

